# Photodynamic Surgery for Feline Injection-Site Sarcoma

**DOI:** 10.1155/2019/8275935

**Published:** 2019-07-08

**Authors:** Marina Martano, Emanuela Morello, Sofia Avnet, Francesco Costa, Federica Sammartano, Katsuyuki Kusuzaki, Nicola Baldini

**Affiliations:** ^1^Department of Veterinary Sciences, University of Turin, 10095 Grugliasco, Italy; ^2^Orthopaedic Pathophysiology and Regenerative Medicine Unit, IRCCS Istituto Ortopedico Rizzoli, Bologna 40136, Italy; ^3^Department of Musculoskeletal Oncology, Tenri 632-000, Japan; ^4^Department of Biomedical and Neuromotor Sciences, University of Bologna, Bologna 40127, Italy

## Abstract

Musculoskeletal sarcomas are rare and aggressive human malignancies affecting bones and soft tissues with severe consequences, in terms of both morbidity and mortality. An innovative technique that combines photodynamic surgery (PDS) and therapy (PDT) with acridine orange has been recently suggested, showing promising results. However, due to the low incidence of sarcoma in humans, this procedure has been attempted only in pilot studies and stronger evidence is needed. Naturally occurring tumors in cats are well-established and advantageous models for human cancers. Feline injection-site sarcoma (FISS) shares with human musculoskeletal sarcomas a mesenchymal origin and an aggressive behavior with a high relapse rate. Furthermore, wide surgical excision is not always possible due to the size and site of development. We assessed the feasibility and the effectiveness of PDS and PDT with acridine orange to prevent FISS recurrence by treating a short case series of cats. For PDS, the surgical field was irrigated with an acridine orange solution and exposed to UV light to enlighten the residual tumor tissue, and the resultant fluorescent areas were trimmed. For PDT, before wound closure, the field was again irrigated with acridine orange solution and exposed to visible light to get the antitumoral cytocidal effect. The procedure was easy to perform and well tolerated, we did not observe any major complications, and all the surgical resection margins were free of disease. Finally, at follow-up, all treated patients did not show evidence of tumor recurrence and had a significantly higher event-free survival rate in respect to a control group treated only by surgery. In conclusion, by this study we demonstrated that, in FISS, PDS and PDT with acridine orange may improve local tumor control, granting a better outcome, and we laid the foundation to validate its effectiveness for the treatment of human musculoskeletal sarcomas.

## 1. Introduction

Musculoskeletal sarcomas are relatively rare human malignancies that often occur in children and young adults, leading to devastating consequences in terms of both morbidity and mortality [1]. Currently, the gold standard therapy is surgery, followed by chemotherapy and/or radiotherapy, but the 5-year survival rate is still quite low [2].

Limb salvage is a well-established technique for the treatment of musculoskeletal sarcomas and the most common approach includes wide resection followed by limb reconstruction [3]. However, when sarcoma arises around the forearm or the hand, involving major nerves and vessels, this technique may cause the sacrifice of noble structures that may lead to important limb disabilities, with a meaningful negative impact on the quality of life.

In this context, we have recently described a novel limb salvage technique based on the use of acridine orange for photodynamic surgery (PDS) that is coupled with photodynamic therapy (PDT) and radiodynamic therapy (RDT) [4–8]. This innovative technique aims at improving surgery by achieving minimally invasive excision margins and by decreasing the number of complications for the treatment of musculoskeletal sarcomas. Acridine orange is a fluorescent cationic dye that is extracted from coal tar and creosote oil. It owns metachromatic properties and various unique biological activities that can be harnessed in different ways: as a fluorescent dye specific for DNA and RNA, as a pH indicator, as a photosensitizer, as an antimalarial drug, as a detector of bacteria and parasites, and, more importantly, as an anticancer agent [9]. Since it has a low molecular weight, acridine orange can easily diffuse into interstitial tissues and cytoplasm of cells to bind cellular structures and organelles. Due to protonation, acridine orange accumulates into intracellular acid vesicles, leading to the formation of membrane-impermeable monomeric, dimeric, or oligomeric aggregates [10–12]. In this context, it is important to note that, as first described by the Nobel Prize laureate Otto Heinrich Warburg in 1924, malignant tumors, including musculoskeletal sarcomas, are highly glycolytic [13, 14]. One of the major consequences of altered glycolytic metabolism is that tumors are more acidic than surrounding tissues [15]. Increased acidification in cancer might also derive from oxidative cancer cells, thus from the production of CO_2_ which is hydrated into HCO_3_^−^ and H^+^ ions by carbonic anhydrases. The pentose phosphate pathway and glutaminolysis also contribute to CO_2_ production [15]. Venting of CO_2_, H^+^, lactate, and/or HCO_3_ is critical to minimize cytosolic acid accumulation. To avoid acidification of intracellular pH, acid produced by tumor cells must be compartmentalized in acid organelles or extruded outside the cell membrane, respectively. A various number of protons and ions pumps and transporters, at both the lysosome and the cytosolic membranes, are implied in this process [16]. However, compromised blood perfusion in tumors complicates intracellular H^+^ removal [15]. It is claimed that, unlike normal cells, sarcoma cells have highly acidic lysosomes and produce significant acidification of the extracellular microenvironment [17–19]. Acridine orange has thus the potential for a high and selective tropism for highly acidifying tumor cells. When illuminated by blue light (466.5 nm) [20], or exposed to low-dose (1-5 Gy) X-ray irradiation [21], acridine orange is cytotoxic through the generation of singlet oxygen (^1^O_2_). The formed reactive species oxidize the fatty acids of the lysosomal membrane, causing the leakage of lysosomal enzymes and protons followed by cell death [12]. We have successfully developed and applied to clinical cases of musculoskeletal sarcoma a combined technique of PDT and RDT based on the use of acridine orange, demonstrating an excellent clinical outcome in terms of inhibiting local recurrence and preserving limb function after intra- or marginal tumor resection [5–7, 22]. This technique proved to be particularly advantageous in sarcomas arising around the forearm and a valid alternative to wide surgical resection followed by limb reconstruction, without increasing the local recurrence rate [23].

Despite the presence of numerous pilot studies concerning the use of acridine orange for the treatment of musculoskeletal sarcomas, the low incidence of these tumors in humans is limiting the development of clinical trials demonstrating the efficacy of this procedure compared to standard treatments. To extend the clinical database and increase the statistical significance of these results, in this study we involved comparative oncology. Naturally occurring tumors in animals, such as cats or dogs, are well-established models for human cancers [24]. Their shorter lifespan is useful for rapid trial completion and data collection, and the lack of standard of care in most cases allows comparative evaluation of new therapies [25].

Feline Injection Site Sarcoma (FISS) is a malignant tumor of mesenchymal origin that develops in 1–10 of 10,000 injected cats. The pathogenesis of this disease is unknown, although the most accepted hypothesis is a hyatrogenic origin due to local postinjection inflammatory response that leads to neoplastic transformation [26]. FISS has histological and behavioral characteristics similar to human musculoskeletal sarcomas, since it shares with these tumours a fibroblast-like histology and an aggressive clinical behavior [26]. Indeed, FISS has a rapid growth with a high tendency to infiltrate the surrounding tissues, to such an extent that a wide surgical excision is not always achievable. Furthermore, as for human musculoskeletal sarcomas, FISS often recurs in spite of wide surgical resection and radiotherapy, which represents the current gold standard of therapy [27–29].

In this study, through comparative oncology, we aimed to establish FISS as an optimum model to consolidate the effectiveness of the combined approach PDS-PDT using acridine orange in reducing postsurgical recurrence rate of human musculoskeletal sarcoma.

## 2. Materials and Methods

### 2.1. Clinical Series

Starting from March 2014 and up to April 2015, we enrolled a short case series that presented for examination at the Veterinary Teaching Hospital of the University of Turin (Grugliasco, Italy) with a growing mass at injection sites. Before any procedure, the Institutional Animal Care and Use Committee approval (11 September 2011) was obtained, and the informed consent clearly describing the technique was signed by the animal owner, clearly describing the technique. After a thorough physical examination, each patient underwent a complete blood count, a biochemical profile, and a blood test for Feline Immunodeficiency and Feline Leukaemia Viruses (FIV, FeLV) and was diagnosed with FISS, as confirmed by fine needle biopsy and histological examination. The history always reported the occurrence of a rapidly growing mass at sites of injections of vaccines or other drugs. All cats underwent total body computed tomography (CT) scan under general anesthesia to estimate the size of the tumor, to determine the area of surgical excision, and to detect lung metastases.

### 2.2. Procedure for Photodynamic Surgery

A standard oncological resection was performed in the sternal decubitus position, with the exception of one case for which the patient was put in lateral decubitus due to the location of the tumor on the lateral aspect of the thorax. The surgical excision was performed according to the FISS treatment guidelines [27], under general anesthesia maintained with isoflurane (Pfizer, NY, USA) in oxygen. En-bloc resection of the tumor was performed based on CT measurement after contrast enhancement, with a 3 cm safety margin, removing skin, subcutaneous tissue, and muscle to the fascial layer below the tumor. Hemostasis was obtained by electrosurgery (MB160, GIMA, Milan, Italy). Blood loss was minimal. After tumor excision, the surgical field was first irrigated with 50 mL sterile saline solution to remove blood clots and then with a 50 mL of a acridine orange (1*μ*g/mL, Sigma Aldrich Co. St. Louis, MO, USA) sterile solution and left in darkness for 10 min. After lavage with sterile saline solution, the surgical field was excited with blue light with two different devices that were suitable for photoactivation in the operating room: Starlight Pro, Mectron®, Genova, Italy (wavelength 488 nm), or Rechargeable Custom Blue Light (wavelength 440/460 nm, LEM srl, Italy). The type of the device was chosen depending on the size of the area. Fluorescent areas were further excised with Metzenbaum scissors, until fluorescence was no longer visible. The whole excised sample, together with the excised residues after exposure with acridine orange, was submitted to histopathology after inking the margins, in order to confirm the diagnosis and evaluate the radicality of excision.

### 2.3. Procedure for Photodynamic Therapy

Soon after the surgical procedure, the field was irrigated with a 50 mL syringe filled with acridine orange sterile solution (1*μ*g/mL) and exposed to surgical light (ML701, KLS Martin, Germany, 24V/250Watt, 80000 Lux) for 10 minutes, immediately after acridine orange irrigation. Finally, the wound was sutured as routine.

### 2.4. Intratumoral pH Evaluation

Immediately after the excision, the tumor pH was assessed (three replicates for each lesion) using a portable pH Level Monitor (Hanna Instruments, Padova, Italy) and recorded.

### 2.5. Follow-Up and Event-Free Survival Rate

After the procedure, animals were hospitalized for 2 days and reassessed after 7 and 15 days and every 3 months thereafter for the first year and every 6 months the second year. At each control, starting at 3 months, chest radiographs were taken to evaluate the development of metastases and a thorough physical examination was performed. After the second year the follow-up was updated by phone call to the owners or the referring veterinarian. As a control, we considered both a retrospective and a prospective group of 30 FISS treated by en-bloc surgery alone, recruited from January 2009 to September 2015. In order to parallel the maximum follow-up time of the treated group, the maximum follow-up considered for both groups was 1444 days from the date of the surgery. The decision to retrospectively recruit cases for the control group was due to the decrease in the number of cats affected by the disease visited in the last years, at least at the institution where the study was conducted. Anyway, the diagnosis and staging of each animal were accomplished in the same way, and the surgical procedure was performed by the same surgeons in both groups. The only variable in the procedure was the PDS-PDT added in the study group. To evaluate the rate of success for the PDS and PDT-treatment, the event-free survival was calculated from the date of surgery to the date of detection of recurrence or metastasis. The average follow-up was 902 ± 84 days for the control group and 961 ± 223 days for the treated group.

### 2.6. Statistical Analysis

Statistical analysis was performed by GraphPad Prism 7 software (GraphPad Software, Inc). Quantitative results were expressed as mean ± standard error (SEM). Due to the low number of events considered in the study, we used nonparametric test. Survival analysis was performed by using Kaplan-Meier survival plot (Log-rank Mantel-Cox test). Only* p* < 0.05 was considered significant. Animals were censored if they were alive and did not show recurrence and/or metastasis at the end of the study, if they were lost at follow-up, or if they died for reasons not related to the tumor.

## 3. Results

### 3.1. FISS Series and PDS Treatment with Acridine Orange

In this study we enrolled seven client-owned cats, while 30 cats were used as control (Table 1).

**Figure 1:**
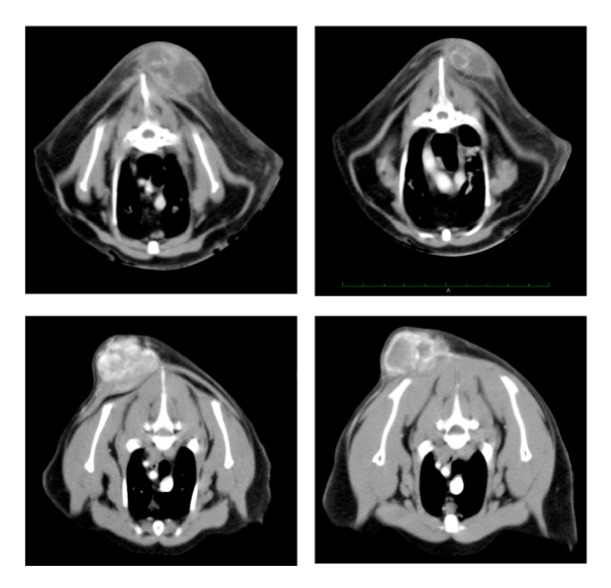
Preoperative CT scan of cats # 2 and 3 of the treated group. The extent of the tumor after contrast medium administration was considered including the lateral laciniae; infiltration into surrounding tissues and the vicinity with spinal processes were assessed.

**Figure 2:**
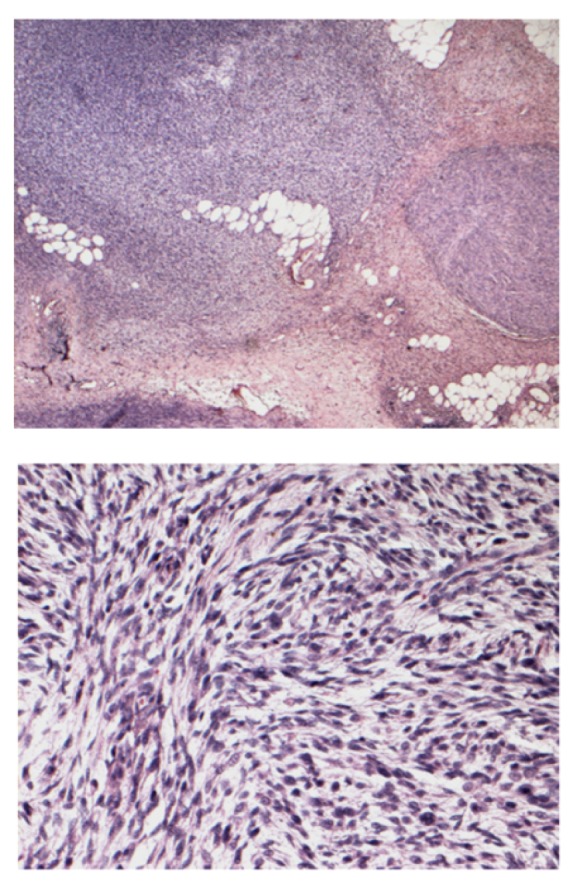
Histopathological image of FISS (Courtesy of dr. S. Iussich). Upper panel, multiple nodules of fusiform cells arranged in sheets in abundant hyaline stroma infiltrating the subcutaneous tissue (H&E, 2x objective); lower panel, multiple nodules of fusiform cells arranged in sheets in moderate myxoid stroma; the cells (20-30 *μ* in diameter) are characterized by scant cytoplasm, ovalar and vesicular nuclei with 1 nucleolus not always visible; low mitotic index (0-1/HPF) (H&E, 20x objective).

The median age of the treated cats was 11 years (min 10 – max 12); for the control group it was 11 years (min 3 – max 15). As revealed by CT scan, the maximum tumor volume, that was calculated according to the formula: tumor volume [cm^3^] = (length [cm] x width^2^ [cm^2^])/2 [30], ranged from 86.2 to 1362.4 mm^3^ (median 193.6 cm^3^) for the treated group and 0.25 to 1316 cm^3^ (median 132 cm^3^) for the control group. Representative CT scans for the treated group are shown in Figure 1 and histology is shown in Figure 2.

After tumor resection, the median pH value was 6.14 (min 6.08, max 6.84, Table 2).

**Figure 3:**
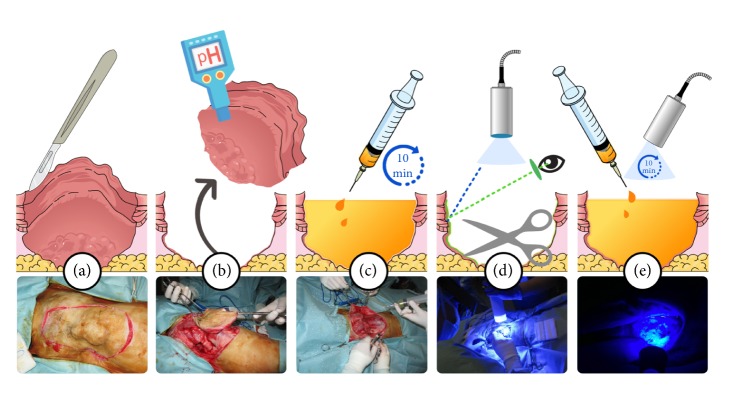
Graphical representation of acridine orange PDS and PDT technique. Surgical excision (a), pH measurement (b), acridine orange irrigation (c), PDS (d), PDT (e). On the bottom line, representative photographs of the procedure are shown.

PDS and PDT are summarized in Figure 3.

Resection of spinous processes of adjacent vertebrae was needed in three cases (#2, 3, and 7). Overall, the surgery time was 30 minutes longer as compared to the standard surgical procedure. No major complications were observed. The procedure was well tolerated by the animals. Tumor-free excision margins were obtained in all cases.

### 3.2. Impact of PDS and PDT on FISS Recurrence Rate

**Figure 4:**
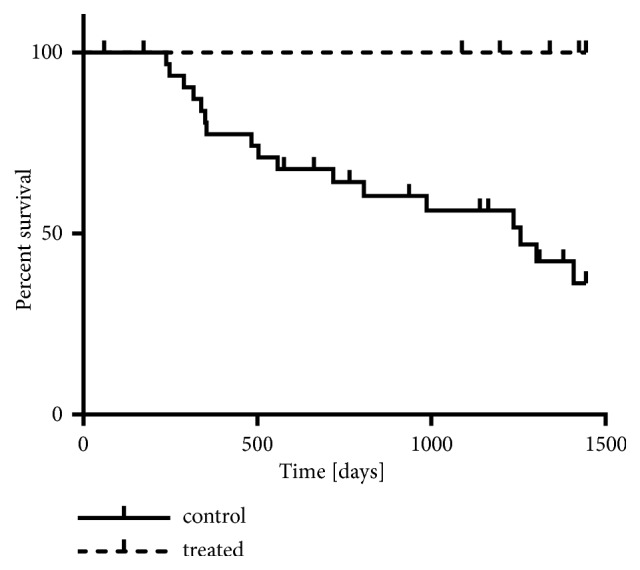
Effect of PDS and PDT with acridine orange on FISS disease-free survival time at follow-up, as analyzed by Kaplan-Meier. Both local recurrence and metastasis are considered as event (n. of uncensored events was 17 for control and 0 for treated, out of 30 and 7 patients, respectively).

Two patients (#3, 6) were euthanized for unrelated causes after 60 and 173 days from surgery (infectious viral peritonitis and chronic bowel disease, respectively), and they did not present evidence of local or systemic relapse of the tumor at the time of death. All remaining cats were alive and did not develop recurrence and/or metastasis at the time of last follow-up. The Kaplan-Meier analysis was applied to estimate the survival rate. Although no significant association was found with local recurrence rate (data not shown), the PDS and PDT treatment was associated with a significantly higher event-free survival rate in comparison with the control group (Figure 4,* p* = 0.0482). 12 out of 30 cases developed local recurrence (40%), and 17 out of 30 developed local recurrence and/or lung metastasis (56.6%).

## 4. Discussion

Intratumoral acidosis is a hallmark of cancer [14–16], and since Otto Warburg's reports in 1925, evidence of a correlation between tumor glycolysis and extracellular acidosis has been demonstrated by several techniques, including acidoCEST MRI and [^18^F]FDG PET [31, 32], suggesting an inverse correlation between extracellular tumor pH and [^18^F]FDG uptake. Other factors, besides increased glycolysis, have been suggested as responsible for the induction of intratumoral acidosis, such as the production of CO_2_ during mitochondrial respiration when combined with a defective proton-venting cellular mechanism [15]. Intratumoral acidosis may be used as a selective anticancer target, and different antiacid therapeutic modalities have been developed, such as neutralization of tumor-derived acid by systemic buffers, proton pump inhibitors, or carbonic anhydrase inhibitors [15, 18, 33, 34]. The use of pH-sensitive drug-delivery nanocarriers has also been proposed [15, 35]. Photodynamic technology may be exploited as an additional anticancer approach for acid microenvironment. Photoactivation involves activating or inactivating specific molecules using light. Light causes the activation, inhibition, or conformational changes in these molecules. As an example, drug photoactivation has been tested for the treatment of cancer, as demonstrated in several types of carcinomas and in glioblastoma [36–40]. In this study, we used PDS and PDT based on acridine orange, a fluorescent cationic dye with notably advantageous features, since it selectively targets acidifying tissue [41], is not tumorigenic in humans, mice, rats, or rabbits [42], and has been suggested for clinical application [4]. Regarding PDS, acridine orange is an innovative tool to increase surgery efficacy, since it avoids the need for wide surgical margins, thereby reducing postsurgery disabilities [43]. Acridine orange gives a further advantage by exploiting of the PDT action since its cytotoxicity is selective against cancer cells. Indeed, this molecule accumulates into lysosomes that are highly acidic, and, when photoactivated, it causes lysosomal membrane permeabilization, by the formation of oxygen single species [12], and the consequent leakage of the lysosomal content into the cytosol, leading to the so-called “lysosomal cell death.” This form of cell death can have necrotic, apoptotic, or apoptosis-like features depending on the extent of the leakage and the cellular context [44]. Malignant transformation is associated with alterations in lysosomal structure and function, which, paradoxically, render cancer cells more sensitive to lysosomal destabilization [45]. Similarly to other types of cancers, sarcoma cells have very acidic lysosomes, especially in the case of cancer stem cells and drug-resistant cells [33, 46].

Despite the preclinical evidence of the potential of acridine orange combined to PDS and PDT to treat sarcomas, the low incidence of these malignancies in humans has not allowed a wide application of such technique. In this context, comparative oncology may greatly help in the evaluation and interpretation of the response to treatment, especially when human and animal diseases are biologically and clinically similar [47], as in the case of sarcomas.

In this pilot study, we enrolled cats with FISS, a soft tissue sarcoma with clinical futures very similar to those of human tumors, including an aggressive local behavior. Up to one-quarter of cats with this condition have metastatic lung involvement, but the major concern is related to the high recurrence rate of the disease. The mainstay of treatment is aggressive surgery, but even in cases of wide excision with clean margins, tumor recurrence occurs in a high proportion of cases [48–51]. The effectiveness of adjunctive chemotherapy is still controversial [29], and radiotherapy is still not widely available in veterinary medicine. In such contest, PDS and PDT may be of great advantage, allowing a thorough excision, thereby reducing the extent of the excision area and postoperative morbidity. PDT has already been studied in FISS, based on the use of indocyanine green combined to hyperthermal chemotherapy following conservative surgical resection [52]. In our series, cats were treated with acridine orange-based PDS and PDT, a treatment already tested in different pilot studies on malignant musculoskeletal tumors in humans [8, 41, 53–55]. Tumor samples showed a low intratumoral pH. As the blood loss during surgery was minimal, the pH measurement was not affected by the buffering activity mediated by bicarbonate in the blood. These data demonstrate that FISS is an acid-producing tumor to an extent similar to those of the most aggressive human sarcomas. In fact, Matsubara et al. have previously reported that intratumoral pH of malignant musculoskeletal tumors is 6.78 ± 0.26 [41], and Engin et al. reported an intratumoral pH value for soft tissue sarcomas of 7.01 ± 0.21[56]. Notably, normal tissues, such as muscle or adipose tissue, have an interstitial pH of 7.26 ± 0.14 and 7.43 ± 0.11, respectively [41].

After the execution of PDS and PDT, we did not observe any adverse event, neither during nor in the immediate follow-up. The procedure was very similar to that previously used in human patients with sarcomas [4, 54], with the only exception that we used surgical loupes in place of a surgical microscope. For the local recurrence rate, although we did observe a trend of reduction after the PDS and PDT treatment, this difference was not significant in respect to the control group, possibly because of the low number of cases enrolled in the study. However, we obtained a significant reduction of tumor relapse, intended as local recurrence or development of lung metastasis, thereby suggesting that the local control might have a positive effect in preventing the formation of metastasis at distant sites. These unexpected results might reflect the occurrence of a bystander effect (abscopal effect) that has been observed after radiation therapy in several types of cancer, including sarcoma [57]. In radioimmunobiologic terms, abscopal effects describe the radiotherapy-induced regression of cancerous lesions distant from the primary site of radiation delivery and rely upon the induction of immunogenic cell death and consequent systemic anticancer immune activation. Similarly, the treatment with PDT might have induced the necrosis of the remaining infiltrated tumor cells after the PDS treatment, close to the tumor resection site, thereby causing the release of tumor-associated antigens that, in turn, might have sensitized and enhanced the host immune surveillance against the systemic residual disease.

The recurrence rate observed in the control group was higher compared to what is reported by the most recent veterinary literature [28]; this was because some of the cats presented with very large tumors that would have greatly benefited from adjuvant radiotherapy, which was declined by the owners. In these cases, wide surgical margins were not achievable, and this may explain the outcome.

## 5. Conclusions

Our data suggest that PDS-PDT based on the use of acridine orange has the potential to exert a similar effect in comparison to the gold standard treatment of FISS and that, according to the results obtained by analyzing a pilot series of animal patients with FISS, this technique may be considered a useful adjuvant therapeutic modality.

Our data lay the foundation for additional research in this spontaneous animal condition that may foster the development of novel treatment options to be translated for human musculoskeletal sarcomas.

## Figures and Tables

**Table 1:** Patients series.

	Case#	Age	Sex	Race	Recurrence	Metastasis	Status at follow-up*∗*	Length of follow-up
treated	1	11	M	Domestic short hair	No	No	censored	1197
treated	2	10	MC	Domestic short hair	No	No	censored	1340
treated	3	12	F	Domestic short hair	No	No	censored	173
treated	4	12	FS	Siamese	No	No	censored	1444
treated	5	10	MC	Domestic short hair	No	No	censored	1424
treated	6	11	FS	Maine Coon	No	No	censored	60
treated	7	12	MC	Domestic short hair	No	No	censored	1088
control	1	6	FS	Domestic short hair	No	No	censored	1444
control	2	10	MC	Domestic short hair	No	No	censored	1444
control	3	9	MC	Domestic short hair	No	No	censored	1444
control	4	8	MC	Domestic short hair	Yes	Yes	uncensored	317
control	5	6	FS	Domestic short hair	No	No	censored	1163
control	6	9	FS	Domestic short hair	Yes	No	uncensored	718
control	7	7	FS	Domestic short hair	Yes	No	uncensored	1302
control	8	8	FS	Domestic short hair	No	yes	uncensored	243
control	9	13	FS	Domestic short hair	Yes	No	uncensored	558
control	10	11	FS	Domestic short hair	No	No	censored	1444
control	11	12	FS	Domestic short hair	No	Yes	uncensored	354
control	12	3	FS	Domestic short hair	No	No	censored	1444
control	13	10	MC	Domestic short hair	Yes	No	uncensored	1236
control	14	12	FS	Domestic short hair	No	Yes	uncensored	339
control	15	11	MC	Domestic short hair	No	No	censored	1444
control	16	10	FS	Domestic short hair	Yes	No	uncensored	350
control	17	13	MC	Domestic short hair	No	No	censored	663
control	18	13	FS	Domestic short hair	No	No	censored	1379
control	19	13	MC	Domestic short hair	Yes	No	uncensored	986
control	20	14	MC	Domestic short hair	Yes	No	uncensored	1256
control	21	15	FC	Domestic short hair	No	No	censored	577
control	22	13	MC	Birmano	No	Yes	uncensored	483
control	23	7	FC	Domestic short hair	No	Yes	uncensored	806
control	24	9	FC	Domestic short hair	Yes	No	uncensored	503
control	25	11	FC	Domestic short hair	Yes	No	uncensored	1409
control	26	11	MC	Domestic short hair	Yes	No	uncensored	238
control	27	11	FC	Domestic short hair	Yes	No	uncensored	289
control	28	13	FC	Domestic short hair	No	No	censored	1310
control	29	13	MC	Domestic short hair	No	No	censored	1139
control	30	14	MC	Domestic short hair	No	No	censored	765

M, male; MC, male castrated; F, female; FS, female spayed; status at follow-up*∗*: censored are cases lost at follow-up, or alive, or dead for causes not related to the tumor without local relapse or metastasis at last follow-up; uncensored are cases that had local recurrence or metastasis at follow-up.

**Table 2:** Intratumoral pH.

Case	Tumour size	pH
#	width x length [cm]	
1	5.0x8.8	n.d.
2	8.5x9.0	6.14
3	9.0x17.4	6.26
4	5.5x5.6	6.08
5	7.3x10.7	6.10
6	6.0x7.0	6.84
7	10.0x5.0	n.d.

n.d., not determined.

## Data Availability

All the data used to support the findings of this study are available from the corresponding author upon request.
